# Book Reviews

**Published:** 1916-07

**Authors:** 


### BOOK REVIEWS

SKIN CANCER. By Henry H. Hazen, A.B., M.D., Professor of Dermatology in the Medical Department of Georgetown University; Professor Dermatology Medical Department of Howard University; Sometime Assistant in Dermatology in Johns Hopkins University; Member of the American Dermatological Association. 251 pages, 97 text Illustrations and a Colored Frontispiece. Cloth, $4.00. St. Louis: The C. V. Mosby Co.

This monograph is well written and copiously illustreated. It covers the field completely and is very timely. The isolation of the consideration of malignancy of the skin from malignancy elsewhere and from other skin troubles is attractive and calls closer attention to many conditions than they usually receive. The fact that an early recognition of these diseases is of great importance in their treatment and contributes largely toward their relief, makes this book of great value of the physician because of its intensive study of skin malignancy.

The chapters are all interesting and the reader can feel equipped to recognize the conditions described whether he is ready to treat them himself or not.

The treatment chapters deal largely with electrical, actinic and radial methods and these, of course, are in the hands of the specialists. All the other methods are described and means of keeping the patients cleanly and comfortable are presented.

The whole work is in close touch with all the recent advances made in the study of cancer and has a practical appreciation of the increase in malignancy that is threatening the people everywhere.

---

DISEASES OF THE SKIN. By Richard L. Sutton, M.D., Professor of Diseases of the Skin, University of Kansas School of Medicine; Former Chairman of the Dermatological Section of the American Medical Association; Member of the American Dermatological Association; Assistant Surgeon United States Navy, Retired; Dermatologist to the Christian Church Hospital. 916 Pages, with 693 Illustrations and 8 Colored Plates. Cloth, $6.50. St. Louis: The C. V. Mosby Company.

This book has the usual attempt at "Classification"—always a difficult problem in cutaneous diseases, though advances in microscopic study of the pathology have cleared up many disputed points. The text is well printed, save for a few typographical errors, and the illustrations are as good on the whole as possible in black and white, and are abundant. The author has done an immense amount of work in getting through the literature, in the acquisition of pictures and in personal correspondence. The index is full and satisfactory. In view of the labor expended and matter condensed, the contraction of the text into a startling brevity is notable. This brevity may detract from the work as a text-book for students, but the Reviewer's use of the book, for a week or two, as a reference, has impressed him with its value to the trained dermatologist. The work seems completely "up to the minute" in this field. Recurring to its value as a text-book, it is probable that students will find it perfection as an adjunct, and source of review, to their didactic and clinical instructions.

Dr. Sutton's knowledge and personal experience are large, his clearness of style and ability to "get at the meat" of his subject is marked, and the book, showing the combination of these elements, is pleasing, practical and commendable.

M. B. H.

---

DISEASES OF THE DIGESTIVE TRACT AND THEIR TREATMENT, By A. Everett Austin, A.M., M.D., Former Professor of Physiological Chemistry at Tufts Colege, University of Virginia and University of Texas; Present Assistant Professor of Clinical Medicine, in charge of Dietectics and Gastrointestinal Diseases, Tufts College; Member of American Gastroenterological Association and American Society of Biological Chemists; Physician to Mt. Sinai Hospital and Berkely Infirmary, and Assistant to Boston Dispensary; Author of "Manual of Clinical Chemistry," etc. With 85 Illustrations, including 10 Color Plates. 552 Pages. Cloth, $5.60. St. Louis: The C. V. Mosby Company.

The writer of this work does not claim originality in its pages, for in his preface he admits that one of his purposes is to "put in a pleasing and useful form that which is already known." A careful scrutiny fails to disclose anything specially new in eithe rdiagnosis of treatment of digestive diseases, thougs the Roentgen diagnosis is covered in a fairly adequate manner. This aspect in diagnosis of disturbances of the alimentary tract is arriving at a more sane and reasonable basis, and the ridiculous claims made for the X-Ray by some hyper-enthusiastic roentgenologists are not voiced by Professor Austain. Aus he claims, the X-Ray has its useful and proper place in the investigation of gastrointestinal maladies, but it has not displaced nor will it set aside the other approved methods.

We regret to note that the writer of this book has seen fit almost exclusively to quote from foreign (mostly German) authorities. The mass of good and intelligent work done by gastroenterologists in this country apparently merits more kindly notice than has been accorded them by Professor Austin.

Though interestingly written, this volume fornishes no special addition to our present knowledge of gastroenterology.

Niles.

Unlike most tonics, Grays Glycerine Tonic Comp. has no contraindications, and can be used with maximum benefit at all seasons and under all conditions.

---

"I have used Tongaline for more than 20 years and have foun dit most satisfactory in every way. A very recent case which came under my care was one in which several physicians had failed, even with the use of organo-therapy. Within 48 hours after Tongaline had been administered, there was a decided remission of temperature and pain and at the end of one month the patient, who was a lady about 70 years of age and had been a sufferer for years, was able to go about her room and to comb her own hair, something which she had not done for 6 months previously."

---

"I prescribed Tongaline for two cases of tonsillitis, after all other treatment had failed, with such success that both made a rapid recovery."

---

### THE PROSPECTIVE MOTHER.

During pregnancy, as a result of the extra tax imposed on every organ, the prospective mother often approaches the critical period of parturiiion without the reserve vitality and strength she obviously needs. To build up her body and reinforce enfeebled or flagging functions is imperative, and here will be shown the great value of Gray's Glycerine Tonic Comp. Used thoughout the later months of pregnancy and following confinement, it gives to the mother the stimulus and support so necessary to enable her to pass successfully through a trying period, and also to meet the still more exacting demands of lactation.

As a result, therefore, of the benefits derived from this dependable tonic and reconstructive, breast feeding is not only made possible, but so greatly facilitated that the family physician cannot fail to appreciate the aid it gives him in his efforts to this end.

